# The roles of aldehyde dehydrogenases (ALDHs) in the PDH bypass of Arabidopsis

**DOI:** 10.1186/1471-2091-10-7

**Published:** 2009-03-25

**Authors:** Yanling Wei, Ming Lin, David J Oliver, Patrick S Schnable

**Affiliations:** 1 Department of Genetics, Development and Cell Biology, Iowa State University, Ames, Iowa 50011, USA; 2 Interdepartmental Genetics Program, Iowa State University, Ames, Iowa 50011, USA; 3 Department of Agronomy, Iowa State University, Ames, Iowa 50011, USA; 4 Center for Plant Genomics, Iowa State University, Ames, Iowa 50011, USA; 5 Current address : Center for Applied Genetic Technologies, University of Georgia, Athens, GA 30602, USA

## Abstract

**Background:**

Eukaryotic aldehyde dehydrogenases (ALDHs, EC 1.2.1), which oxidize aldehydes into carboxylic acids, have been classified into more than 20 families. In mammals, Family 2 ALDHs detoxify acetaldehyde. It has been hypothesized that plant Family 2 ALDHs oxidize acetaldehyde generated via ethanolic fermentation, producing acetate for acetyl-CoA biosynthesis via acetyl-CoA synthetase (ACS), similar to the yeast pathway termed the "pyruvate dehydrogenase (PDH) bypass". Evidence for this pathway in plants has been obtained from pollen.

**Results:**

To test for the presence of the PDH bypass in the sporophytic tissue of plants, Arabidopsis plants homozygous for mutant alleles of all three Family 2 ALDH genes were fed with ^14^C-ethanol along with wild type controls. Comparisons of the incorporation rates of ^14^C-ethanol into fatty acids in mutants and wild type controls provided direct evidence for the presence of the PDH bypass in sporophytic tissue. Among the three Family 2 ALDHs, one of the two mitochondrial ALDHs (ALDH2B4) appears to be the primary contributor to this pathway. Surprisingly, single, double and triple ALDH mutants of Arabidopsis did not exhibit detectable phenotypes, even though a Family 2 ALDH gene is required for normal anther development in maize.

**Conclusion:**

The PDH bypass is active in sporophytic tissue of plants. Blocking this pathway via triple ALDH mutants does not uncover obvious visible phenotypes.

## Background

Aldehydes vary in length and in characteristics of their alkyl chains but all are usually deleterious to biological systems due to their chemical reactivity. Aldehyde dehydrogenases (ALDHs, EC 1.2.1) oxidize aldehydes into carboxylic acids, using NAD^+ ^or NADP^+ ^as a co-factor. As such ALDHs play an important role in detoxifying aldehydes that are generated endogenously or introduced from the environment. ALDHs are very diverse in that some only use either NAD^+ ^or NADP^+ ^as the co-factor, while others can use both, some oxidize only a limited number of aldehydes, while others have broader substrate spectra, and ALDHs exist in various subcellular compartments, including the cytosol, mitochondria, plastids and microsomes.

Over 550 ALDH genes have been identified across virtually all species, and those from eukaryotes have been classified into more than 20 families [1]. Family 2 ALDHs are mitochondrial or cytosolic homotetrameric enzymes. The well studied human mitochondrial Family 2 ALDH, ALDH2, detoxifies acetaldehyde generated via alcohol intake [2]. Family 2 ALDHs in plants have gained attention since the cloning of *rf2a *gene, a nuclear restorer gene for cytoplasm male sterility in maize, which encodes a mitochondrial Family 2 ALDH, RF2A [3]. Although the molecular mechanisms associated with the restorer function of the *rf2a *gene remain to be resolved, several studies in maize and other species have provided clues as to the physiological functions of Family 2 ALDHs in plants.

Consistent with the physiological function of human Family 2 ALDH in detoxifying acetaldehyde, Liu and Schnable [4] demonstrated that acetaldehyde is one of the best substrates *in vitro *for RF2A, based on the ratio of *K*_cat _to *K*_m_. In addition, one of the mitochondrial Family 2 ALDHs in rice may be responsible for efficient detoxification of acetaldehyde during re-aeration after submergence of rice plants [5,6]. These studies all suggest a role of Family 2 ALDHs during ethanolic fermentation, which is catalyzed by pyruvate decarboxylase (PDC) and alcohol dehydrogenase (ADH) and generates acetaldehyde and ethanol. The detoxification of acetaldehyde by ALDH produces acetate. In yeast and mammals, it has been established that acetyl-CoA synthetase (ACS) can utilize acetate to synthesize acetyl-CoA, both in the mitochondria [7,8] and in the cytosol [9,10].

The *Arabidopsis thaliana *ACS is targeted to the plastid and is encoded by a single gene [11-13]. In plastids acetyl-CoA is utilized for *de novo *fatty acid biosynthesis. Although the acetyl-CoA pool generated by ACS from acetate seems redundant for fatty acid biosynthesis, ACS is hypothesized to play a specialized role in certain cells and tissues [11]. Besides, the redundancy of acetyl-CoA pool from ACS observed above might be due to the low concentration of acetate, probably 0.05 mM (less than one third the *Km *of this enzyme) [13,14]. Indeed, the feeding of radio-labeled acetate indicates that isolated plastids can use exogenous acetate for fatty acid synthesis [15]. To understand how and when ACS contributes to the acetyl-CoA pool of plastids, the study of acetate biosynthesis would be the key, which is readily diffusible across membranes. One potential pathway would be through ALDH utilizing acetaldehyde generated via ethanolic fermentation by PDC from pyruvate or by ADH from ethanol.

**Figure 1:**
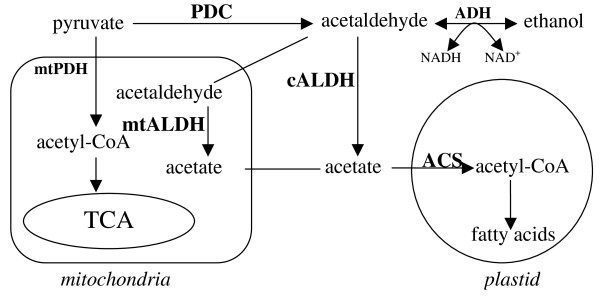
**The PDH and PDH bypass pathways**. PDH, Pyruvate Dehydrogenase; PDC, Pyruvate Decarboxylase; ACS, Acetyl-CoA Synthetase; ADH, Alcohol Dehydrogenase.

In yeast, the PDC-ALDH-ACS pathway (Figure 1) is termed the pyruvate dehydrogenase (PDH) bypass and is used to generate acetyl-CoA involving both cytosolic and mitochondrial ALDHs [16], predominantly when PDH is mutated. [17]. In plants, studies conducted in the Kuhlemeier lab have demonstrated that high expression of PDC [18] and ALDH [19] coincides with high rates of ethanolic fermentation in tobacco pollen. [18]. They supplied growing pollen tubes with ^14^C-ethanol and found label incorporated into fatty acids, indicating that ethanol can be used for fatty acid biosynthesis [20], presumably via the ADH-ALDH-ACS pathway. To provide direct evidence for the presence of the PDH bypass in plants, this study utilized a reverse genetics approach in Arabidopsis to test which (if any) ALDHs are involved in the flux from ethanol into fatty acids.

There are three Family 2 ALDHs in Arabidopsis [21], one of which, ALDH2C4 (AT3G24503), is localized to the cytosol. One physiological function of ALDH2C4 is the production of ferulic acid and sinapic acid during lignin biosynthesis [22]. The other two ALDHs, ALDH2B4 (AT3G48000) [23,24] and ALDH2B7 (AT1G23800) [25], are targeted to mitochondria. Their physiological functions have not yet been described.

## Results

### Identification of null mutants of the three Family 2 ALDH genes

*aldh2B4-1 *and *aldh2B7-1 *T-DNA knockout alleles were identified from the Arabidopsis Knockout Facility at the University of Wisconsin. *aldh2C4-1, aldh2C4-2*, and *aldh2B4-2 *T-DNA knockout alleles were identified from the Salk Institute T-DNA insertion library database. T-DNA insertion site of each line was confirmed via PCR using a T-DNA left border primer coupled with a gene specific primer, either upstream or downstream of the insertion (Table 1).

**Table 1:** Gene-specific primers used along with T-DNA left border primer^a ^for genotyping

	**T-DNA location**	**Gene Specific Primers**		**Insertion feature**^b^
			
		**Forward**	**Reverse**	
*aldh2C4-1 *(SALK_027911)	Exon 3	5'-GCACAACTACTCA TTTTTTTCT-3'	5'-TTGCGGCTGCGGC TTGCATTATCT-3'	F/R

*aldh2C4-2 *(SALK_024974)	Exon 5	5'-TTGATGCGGTTGA CGGTGGAAAAT-3'	5'-AACTTCTCCACAA CCTTATCGTAT-3'	F

*aldh2B4-1 *(CSJ2971)	Intron 7	5'-GTTGGTCCTGCTC TTGCTTGTGGTAA-3'	5'-TCGTTCGCCCTCT TTATCACCTCATC-3'	R

*aldh2B4-2 *(SALK_078568)	Intron 1	5'-ATTCAAAGTACGG CAACACAAACCAAGAG-3'	5'-TTACCACAAGCAA GAGCAGGACCAAC-3'	F/R

*aldh2B7-1 *(CSJ989)	Exon 7	5'-TTGAGACTTGGGA TAATGGGAAACCT-3'	5'-AAGAAAACTGTG ACGGTAATAATCGG-3'	F

The T-DNA insertion sites of mutant alleles were determined by sequencing. However as a consequence of the imprecision of T-DNA integration, it is not possible to establish the exact insertion positions. Only exon/intron positions of the insertions are determined. ^a ^JL202 was used for lines from the University of Wisconsin and LBa1 for those from the SALK institute. ^b ^"F" and "R" indicate amplification of insertion alleles using forward and reverse primers, respectively, in combination with T-DNA left border primer. "F/R" indicate amplification with both primer combinations.

**Figure 2:**
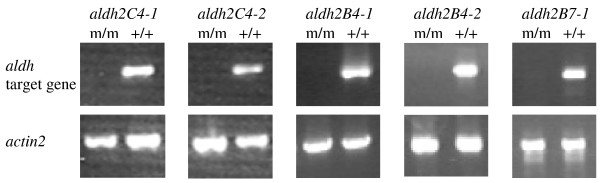
**Mutants used in this study do not accumulate detectable levels of ALDH transcripts**. RNA samples extracted from whole plants were subjected to reverse-transcription PCR using primers specific for the indicated genes (Methods). m/m and +/+ designate RNA samples from plants homozygous for the mutant and wild type alleles, respectively, of the indicated genes. The *actin2 *gene serves as a positive control for RNA quality.

All of the above alleles are null in that homozygous mutants do not accumulate transcripts detectable by reverse-transcription PCR using template RNA isolated from whole plants, while similar template from wild type control plants yielded detectable transcript (Figure 2).

### Direct evidence for the presence of PDH bypass for acetyl-CoA biosynthesis

An ideal experiment to directly test for the presence of the PDH bypass (the PDC-ALDH-ACS pathway) in plants would be to compare the differences between wild type and mutants of involved genes in the incorporation of ^14^C-pyruvate or ^14^C-acetaldehyde into acetyl-CoA in plants in which the PDH pathway has been blocked. This is not possible for two reasons. First, blocking the Arabidopsis PDH pathway by mutating the E2 subunit of the PDH complex results in lethality [12]. Second, pyruvate is unstable and acetaldehyde is toxic. Hence, instead ^14^C-ethanol was fed to plants that were wild type for the PDH pathway and either wild type or mutant for the ALDH genes. The substitution of ethanol for pyruvate or acetaldehyde is appropriate because ethanol should be predominantly, if not exclusively, oxidized to acetaldehyde via ADH (Figure 1). Because well established means to detect acetyl-CoA do not exist, flux through the PDH bypass was measured by determining the incorporation of ^14^C-ethanol into extracted saponifiable lipids, which should consist primarily of fatty acids [13].

^14^C-ethanol was fed to whole seedlings, seedling leaves, and inflorescences. The incorporation rates were compared between single or double *aldh *mutants and their wild type siblings. No differences were detected in whole seedlings or seedling leaves between the *aldh2C4 *homozygous mutants from either allele (*aldh2C4-1 *and *aldh2C4-2*) and their wild type siblings (data not shown). We, therefore, conclude that the cytosolic Family 2 ALDH is either not involved in the ADH-ALDH-ACS pathway, or can be compensated for by the mitochondrial paralog(s).

**Figure 3:**
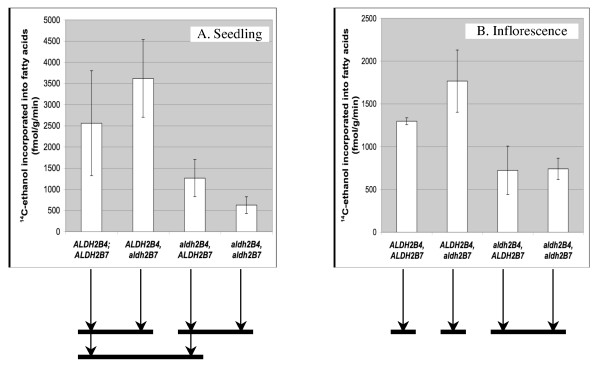
**Incorporation rates of ^14^C-ethanol into fatty acids in whole seedlings (A) and inflorescences (B)**. Reported values are based on the averages of four biological replicates. Each replicate consisted of the indicated tissue from a single plant. The bars under the figures illustrate the results from Tukey's tests, which yielded the same results for the α = 0.05 and the α = 0.1 levels of statistical significance. Genotypes that are connected by a bar are not significantly different from each other (i.e., having p-values > 0.1). In contrast, those that are not connected by a bar are significantly different from each other (i.e., having p-values < 0.05).

Comparisons were also made using all three tissue sources from the *aldh2B4 *single mutant (*aldh2B4;ALDH2B7*), the *aldh2B7 *single mutant (*ALDH2B4;aldh2B7*), the *aldh2B4;aldh2B7 *double mutant and their wild type siblings (*ALDH2B4;ALDH2B7*), all of which were in a uniform WS genetic background (Methods). No statistically significant differences in rates of incorporation were observed in seedling leaves among any of the examined genotypes (data not shown). In contrast, statistically significant differences were observed in whole seedlings and inflorescences (Figure 3). Generally, the comparative results among the four genotypes obtained from whole seedlings and inflorescences are similar. In both whole seedlings and inflorescences, the double mutant of the two mitochondrial ALDHs (*aldh2B4;aldh2B7*) exhibits statistically significant differences relative to the wild type. This demonstrates the role of the mitochondrial ALDHs in the incorporation of ethanol and provides direct evidence for the presence of the PDH bypass in sporophytic tissue (i.e., seedlings).

ALDH2B4 appears to play a more substantial role in this pathway than does ALDH2B7. This conclusion is based in part on the observation that in both tissues the *aldh2B4 *single mutant exhibits less incorporation than the wild type. In addition, in both tissues, the rate of incorporation is not statistically different in the double mutant than in the *aldh2B4 *single mutant. Hence, there is no evidence to indicate that ALDH2B7 contributes substantially to this pathway. Surprisingly, in both tissues, the *aldh2B7 *single mutant (*ALDH2B4;aldh2B7*) exhibits more incorporation than the wild type, but only in the inflorescence is this difference statistically significant. This may indicate that in the *aldh2B7 *mutant, the plant responds by increasing the expression of ALDH2B4, but this hypothesis remains to be tested.

### Expression analyses of Family 2 ALDH genes

**Figure 4:**
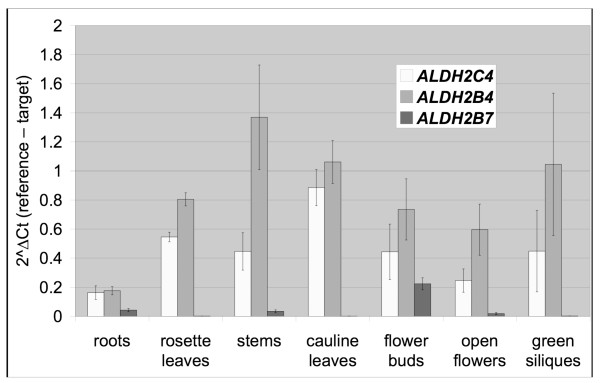
**Expression patterns of Family 2 ALDH genes in Arabidopsis across different tissues**. Three biological replicates were included in the experiment for each tissue, each replicate from an individual plant.

**Figure 5:**
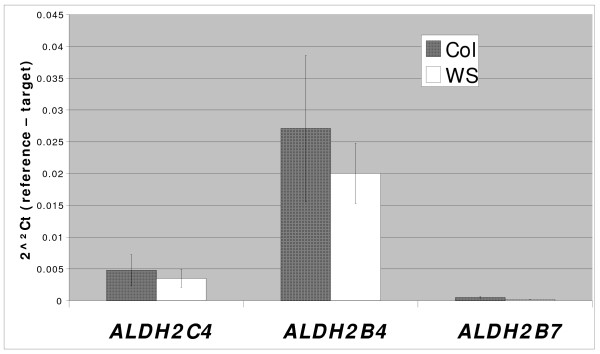
**Transcript levels of the three Family 2 ALDH genes in Arabidopsis whole adult plants of Columbia and WS wild type**. The standard errors indicated by the error bars were calculated by taking three or four primer pairs as replicates for each gene, four for *ALDH2C4*, four for *ALDH2B4 *and 3 for *ALDH2B7 *(Table 2 and Additional File 1).

To understand the expression patterns of the three Family 2 ALDH genes, quantitative Real-Time PCR (qRT-PCR) was conducted on RNA extracted from different tissues from adult wild-type WS plants. These analyses indicate that *ALDH2B4 *and *ALDH2C4 *have similar expression patterns, i.e. they are both constitutively expressed in roots, rosette leaves, stems, cauline leaves, flowers and green siliques, with varying levels across tissues; *ALDH2B7 *has a different expression pattern, i.e., it is predominantly expressed in flower buds compared to other tissues (Figure 4). qRT-PCR from whole wild-type plants with three to four primer pairs for each gene (Table 2 and Additional File 1) showed that in both Columbia and WS ecotypes, *ALDH2B4 *has a much higher expression level than *ALDH2C4 *and *ALDH2B7*, with *ALDH2B7 *the lowest (Figure 5), which is also true for most tissue types.

**Table 2:** Gene specific primers used for qRT-PCR

	**Forward**	**Reverse**
*ALDH2C4*	5'-GATCAACACGGTTTCGAGGT-3'	5'-GCATAACGACGGATTTGGTT-3'
	
	5'-GATCAACACGGTTTCGAGGT-3'	5'-ACATCCAAGGGGAATTGTGA-3'
	
	5'-GAACCAATTGGAGTGGTTGG-3'	5'-GTTGAGCACACCATCAGGAAT-3'
	
	5'-GAACCAATTGGAGTGGTTGG-3'	5'-CCGCTTCTTTTGAGAGATGG-3'

*ALDH2B4*	5'-CTTTTTCAGCTTCCTCTCCC-3'	5'-TGATGAGGAGCTGTGTGTGAG-3'
	
	5'-TGGACAGATCATACCGTGGA-3'	5'-GCATAGAAAGCCGTGAGAGG-3'
	
	5'-TGGACAGATCATACCGTGGA-3'	5'-AGACCCGCTTCAAGGAAAAG-3'
	
	5'-AACAGGGTTTCAAGGGCTTT-3'	5'-GTGACGACTGCCTTGATCTG-3'

*ALDH2B7*	5'-ACCAGCTTTAGCTTGCGGTA-3'	5'-TAGCCCCAAATCCAGAAACT-3'
	
	5'-CGCTCTTTCATGTCCTCCTC-3'	5'-CAACGAATCTTCCACCGATT-3'
	
	5'-GGTACGGTTTAGCTGCTGGA-3'	5'-CCCTCCAAATGGAATTGATG-3'

### ALDH mutants do not exhibit obvious visible phenotypes

No obvious visible phenotypes, including total seed production, plant size, or flowering time, were observed to be associated with any of the single, double or triple ALDH mutants when grown in soil or on MS media. These genotypes were also subjected to several stress conditions, but again no obvious phenotypic differences were observed between mutant and wild type plants. Tested conditions included oxidative stress (4 × 10^-5 ^M Rose Bengal in MS media), ABA treatment (10^-5 ^M in MS media), acetaldehyde (20 mM in MS media) or ethanol (0.5% in MS media), cold treatment (4°C in the dark for 24 hours), heat treatment (40°C in the dark for 12 hours), hypoxia treatments (plants on MS media plates submerged in water in the dark for 6 hours).

## Discussion

The PDC-ALDH-ACS pathway is termed the PDH bypass in yeast [16]. Studies conducted in the Kuhlemeier lab [20] demonstrated that ethanol fed to tobacco pollen can be incorporated into fatty acids, which suggested the presence of the PDC-ALDH-ACS pathway for generating acetyl-CoA for *de novo *fatty acid biosynthesis. Analysis of a PDC mutant of petunia provided further support for the expression of the PDH bypass in pollen [26]. This study left open, however, the question of whether the PDH bypass functions in sporophytic tissue. We used a reverse genetics approach to compare the incorporation rates of ^14^C-ethanol into fatty acids in sporophytic tissue (seedlings) of Arabidopsis *aldh *mutants versus their wild type controls. This study and that of Lin and Oliver [27], which demonstrated lower incorporation rates in an ACS knockout mutant than wild type, provide direct evidence for the presence of the PDH bypass in sporophytic tissue of plants. Specifically, lower rates of incorporation were observed in the *aldh2B4;aldh2B7 *double mutant as compared to wild type controls, in both whole seedlings and inflorescences.

The *aldh2C4 *single mutant did not exhibit differences in incorporation relative to wild type. Because ALDH2C4 can oxidize acetaldehyde, at least *in vitro *[25], this result suggests either that ALDH2C4 is not required for the PDH bypass or that it is involved in the PDH bypass but its absence can be compensated for by the presence of one or both of the mitochondrial ALDHs. Of the two mitochondrial ALDHs, ALDH2B4 appears to play a more important role in the PDH bypass pathway in both whole seedlings and inflorescences.

This study establishes the presence of the PDH bypass in whole seedlings, thereby demonstrating that the PDH bypass is not exclusive to pollen [20]. This conclusion is consistent with our qRT-PCR results, which show constitutive expression of *ALDH2B4. *On the other hand, the findings that a knockout of ALDH2B4 has no discernable phenotype and that an ACS knockout mutant has only modest effects on plant growth [27] suggests that the PDH bypass is not essential under tested conditions. It is not clear whether the PDH bypass is involved in the biosynthesis of acetyl-CoA or the removal of fermentation intermediates. Lin and Oliver [27] provided evidence for the latter in that their ACS knockout mutant is more sensitive to ethanol, acetaldehyde and acetate than are controls. Surprisingly, there are no obvious visible phenotypes associated with any of the single, double or triple Family 2 ALDH mutants, even though a Family 2 ALDH has an essential role in the development of maize anthers [4].

## Conclusion

The PDH bypass, which has previously been shown to be expressed in pollen, can produce acetyl-CoA in the absence of pyruvate dehydrogenase (PDH). We provide evidence of the existence of the PDH bypass in sporophytic tissue. Surprisingly, no obvious visible phenotypes are associated with the triple ALDH mutants, which should block this pathway.

## Methods

### Growth of plants

Wild type and T-DNA insertion lines of Arabidopsis seeds were planted in soil. After planting, they were allowed to imbibe for 2–4 days at 4°C before transferred to 24 hour light conditions in a growth chamber at 22°C under a light intensity of 110 ± 5 μmol m^-2 ^S^-1^.

### Identification and genotyping of T-DNA knockout lines

*aldh2B4-1 *(CSJ2971) and *aldh2B7-1 *(CSJ989) T-DNA knockout lines were identified in association with the Arabidopsis Knockout Facility at the University of Wisconsin following their standard procedures. *aldh2C4-1 *(SALK_027911), *aldh2C4-2 *(SALK_024974), and *aldh2B4-2 *(SALK_078568)T-DNA knockout lines were identified in the Salk Institute T-DNA insertion library database  by BLAST searches.

The T-DNA left border primers JL202 (5'-CATTTTATAATAACGCTGCGGACATCTAC-3') and LBa1 (5'-TGGTTCACGTAGTGGGCCATCG-3') were used in combination with gene specific primers to genotype lines from the University of Wisconsin and from the SALK Institute, respectively (Table 1). LBa1 was used instead of LBb1 (see SALK website) because under our PCR conditions LBb1 can self amplify a band of ~450 bp from Columbia and WS wild type plants.

### Generation of double and triple mutants

To obtain double mutants, crosses were made between plants homozygous for different single mutants to generate a plant that was heterozygous for both. Progeny from this plant were then genotyped. The *aldh2B4;aldh2B7 *double mutant in the uniform WS genetic background carried the *aldh2B4-1 *and *aldh2B7-1 *alleles, and that in a mixed Columbia and WS background carried the *aldh2B4-2 *and *aldh2B7-1 *alleles. The two types of triple mutants carried the *aldh2B4-1, aldh2B7-1 *and one of the two *aldh2C4 *alleles.

### Polymerase Chain Reaction (PCR)

Each PCR reaction included 0.2 mM dNTP, 2.0 mM MgCl_2_, 0.5 μM of each primer, and *Taq *polymerase in a total volume of 20 μl. PCR reactions were conducted for 32 cycles, with each cycle conducted at 94° for 30 sec, followed by the appropriate annealing temperature for 45 sec, and then extended at 72° for 1 min.

### ^14^C feeding and isolation of fatty acids

Per replication, one intact seedling (2 to 4 weeks old) removed from the soil with roots cleaned using a paper towel, one seedling leaf, or the top cluster of flower buds and opened flowers from the main branch of an inflorescence, was weighed, and placed into a 1.5 ml microfuge tube containing 100 μl of carrier (20 mM ethanol) plus 1 μCi of ^14^C-ethanol (Sigma product # 312975). Tubes were incubated in a growth chamber (22°C; 110 ± 5 μmol m^-2 ^S^-1 ^light intensity) with the lid open. After four hours plant tissues were dried with a paper towel and placed into 1 ml Hexane:Isopropanol (3:2) solution for short-term storage if needed. Fatty acids were isolated using the protocol of [13]. Data were collected from four replications.

### Tissue collection, RNA isolation and reverse-transcriptase PCR

Tissues were collected from one month old adult plants growing in soil in the growth chamber as described above, harvested in the following order: green siliques, open flowers, flower buds, cauline leaves, stems, rosette leaves and roots (washed by water and dried with paper towel). After harvest, samples were immediately submerged in liquid nitrogen and stored at -80°C until RNA isolation was performed.

Tissues were ground with a mortar and pestle in liquid nitrogen, and RNA was isolated with a modified "acid guanidinium thiocyanate-phenol-chloroform extraction" method [28,29] as described in [30], except for a slightly different recipe for Trizol (38% phenol equilibrated pH 4.3, 1 M guanidine thiocyanate, 1 M ammonium thiocyanate, 0.1 M sodium acetate pH5, 5% glycerol).

First strand cDNA was synthesized with poly dT primer using SuperScript II RNase H Reverse Transcriptase (Cat. No. 18064-014, Invitrogen, CA).

Two microliters of the first strand cDNA were used for PCR to test the transcription of the T-DNA insertion alleles. All primer pairs flank at least one intron.

The following pair of primers was used for *aldh2C4-1*and *aldh2C4-2*: 5'-AACTTCTCCACAACCTTATCGTAT-3' (forward) and 5'-ACGGAGCCACGACGGTGAAGTTAC-3' (reverse).

The following pair of primers was used for *aldh2B4-1*and *aldh2B4-2*: 5'-CTACTGGATGTGCCTGAAGCATC-3' (forward) and 5'-CATGAGTCTTTAGAGAACCCAAAG-3' (reverse).

For *aldh2B7-1*, the primer sequences are 5'-AGTACCAATGCTTGCTAGGG-3' (forward) and 5'-AGCTTGTAATGTGGCTCCAG-3' (reverse).

The primer sequences used for the positive control *actin2 *(GenBank accession no. U37281) are the same as used by [31].

### Quantitative Real-Time PCR (qRT-PCR)

Procedures similar to those described in [32] were used. The criteria for designing primers (Table 2) using Primer 3 [33] were as follows: Tm, 58°C to 61°C, no difference >2°C between the primers in a pair; primer length, 19–24 bp; GC content, 45–55%; amplicon length, 100–200 bp. Only primers yielding a single product in conventional PCR and qRT-PCR were used. qRT-PCR was conducted by using an Mx4000 multiplex quantitative PCR system (Stratagene). A human gene (GenBank accession no. AA418251) was spiked into each reaction as an external reference for data normalization.

qRT-PCR data were initially analyzed by using MX4000 analysis software. Ct values for each target gene and reference gene were calculated by using baseline-corrected, ROX-normalized parameters. Three technical replicates were included in each plate, and the average Ct value for each gene of interest was normalized within a plate to the human reference gene by computing 2^ΔCt (reference – target) [34] to indicate the relative amount of expression level compared to the reference gene. This was normalized again by the amount difference of starting RNA. The 2^ΔCt (reference – target) values from three biological replicates were used to calculate standard errors.

## Authors' contributions

YL conducted the bulk of the experimental work, interpreted results, and drafted the manuscript. ML participated in the ^14^C-ethanol feeding experiments. DO participated in the design of the ^14^C-ethanol feeding experiments and the interpretation of the results. PS conceived the study, and participated in its design, coordination and interpretation, and helped to draft the manuscript. All authors read and approved the final manuscript.

## Supplementary Material

Additional file 1 5 Introns are represented by lines, exons by black boxes, UTRs by grey boxes. T-DNA insertions are represented by triangles. If a triangle is divided into halves by a line in the middle, the corresponding allele has both the left and the right T-DNA borders. Paired primers used for qRT-PCR are connected by dashed lines. Click here for file
