# Supplementary figures and images for: The roles of aldehyde dehydrogenases (ALDHs) in the PDH bypass of Arabidopsis
Source: BMC Biochem. 2009 Mar 25;10:7. doi: 10.1186/1471-2091-10-7 (PMC2670319; doi:10.1186/1471-2091-10-7)

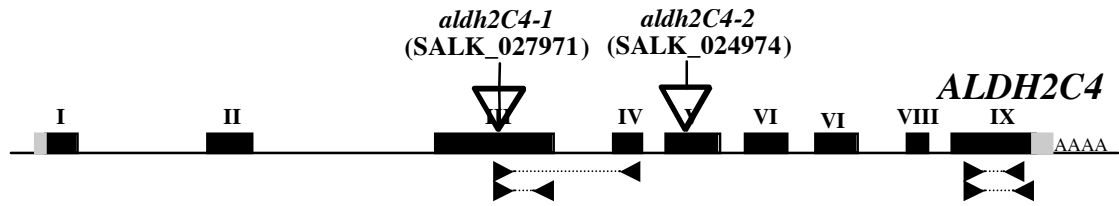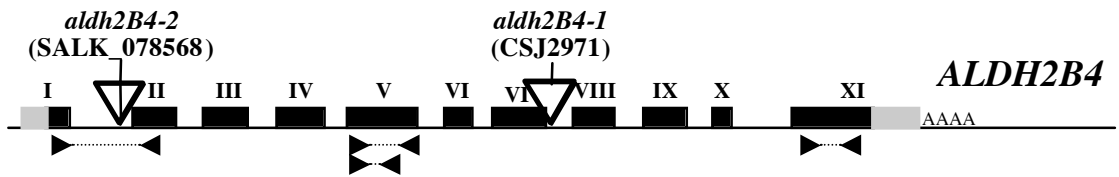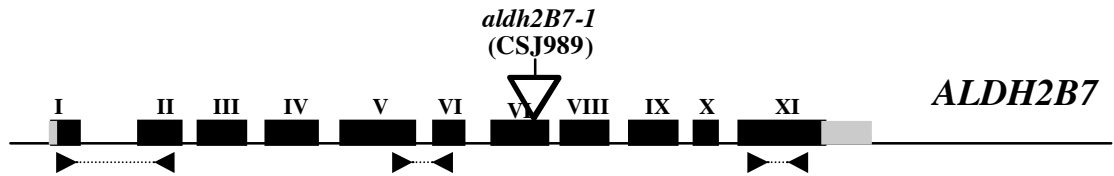

Supplement: Additional file 1 — 5 Introns are represented by lines, exons by black boxes, UTRs by grey boxes. T-DNA insertions are represented by triangles. If a triangle is divided into halves by a line in the middle, the corresponding allele has both the left and the right T-DNA borders. Paired primers used for qRT-PCR are connected by dashed lines. [file 1471-2091-10-7-S1.pdf]
